# A comparative proteomic study identified calreticulin and prohibitin up-regulated in adrenocortical carcinomas

**DOI:** 10.1186/1746-1596-8-58

**Published:** 2013-04-15

**Authors:** Ming-shan Yang, Huan-sheng Wang, Bao-sheng Wang, Wan-hu Li, Zeng-fen Pang, Ben-kui Zou, Xin Zhang, Xue-tao Shi, Dian-bin Mu, De-xian Zhang, Yong-sheng Gao, Xiao-wen Sun, Shu-jie Xia

**Affiliations:** 1 Department of Urology, Shandong Tumor Hospital, 440 Jiyan Road, Jinan 250117, Shandong Province, China; 2 Department of Pathology, Shandong Tumor Hospital, Jinan, Shandong 250117, China; 3 Department of Urology, Shanghai First Hospital, Shanghai 10080, China

**Keywords:** Adrenocortical carcinomas, Proteomics, Tumor marker, Calreticulin, Prohibitin

## Abstract

**Background:**

Identifying novel tumor biomarkers to develop more effective diagnostic and therapeutic strategies for patients with ACC is urgently needed. The aim of the study was to compare the proteomic profiles between adrenocortical carcinomas (ACC) and normal adrenocortical tissues in order to identify novel potential biomarkers for ACC.

**Methods:**

The protein samples from 12 ACC tissues and their paired adjacent normal adrenocortical tissues were profiled with two-dimensional electrophoresis; and differentially expressed proteins were identified by mass spectrometry. Expression patterns of three differently expressed proteins calreticulin, prohibitin and HSP60 in ACC, adrenocortical adenomas (ACA) and normal adrenocortical tissues were further validated by immunohistochemistry.

**Results:**

In our proteomic study, we identified 20 up-regulated and 9 down-regulated proteins in ACC tissues compared with paired normal controls. Most of the up-regulated proteins were focused in protein binding and oxidoreductase activity in Gene Ontology (GO) molecular function classification. By immunohistochemistry, two biomarkers calreticulin and prohibitin were validated to be overexpressed in ACC compared with adrenocortical adenomas (ACA) and normal tissues, but also calreticulin overexpression was significantly associated with tumor stages of ACC.

**Conclusion:**

For the first time, calreticulin and prohibitin were identified to be novel candidate biomarkers for ACC, and their roles during ACC carcinogenesis and clinical significance deserves further investigation.

**Virtual slides:**

The virtual slides for this article can be found here: http://www.diagnosticpathology.diagnomx.eu/vs/1897372598927465

## Background

Adrenocortical carcinomas (ACC) is an extremely rare malignancy, accounting for 0.2% of cancer deaths annually in the world [1]. Most of ACC are carcinomatous, an extremely minor proportion of ACC tumors are characterized by the presence sarcomatous components [2]. Recently, a couple of molecular pathways such as Wnt/β-catenin signaling have been found to be involved in the carcinogenesis of ACC [3]. However, for the lack of early detection markers and effective treatments, ACC patients, especially advanced-stage patients still have a poor prognosis [4].

Several recent studies have identified a panel of novel biomarkers or potential targets for ACC. For example, Sbiera et al. [5] found that survivin overexpression was associated with a poor prognosis for ACC patients, and targeting survivin might be an interesting new therapeutic approach for ACC. Gaujoux S, et al. [6] confirmed that the presence of β-catenin nuclear staining is an independent prognostic factor of overall and disease-free survival in patients with resected primary ACC. Duregon et al. [7] suggest that detection of steroidogenic factor 1 (SF-1) could be a novel prognostic marker in adrenocortical cancer. Insulin growth factor type 2 has been demonstrated consistently overexpressed in ACC, and targeting its receptor IGF1R has shown encouraging treatment potential [8]. However, candidate biomarkers for ACC are relatively limited compared with other cancer types. Therefore, there is still an urgent need to identify novel tumor biomarkers to develop more effective diagnostic and therapeutic strategies for patients with ACC.

The comparative proteomic strategies provide useful tools in identifying novel biomarkers for multiple cancers. According to our knowledge, until now, there is still no proteomic study reported on ACC samples. In this study, we compare the proteomic profiles of ACC tissues and their paired normal adrenocortical tissues by two-dimensional electrophoresis (2-DE) and tandem mass spectrometry. A panel of proteins aberrantly expressed in ACC tissues were identified, and part of them were further validated by immunohistochemistry in a larger cohort of samples.

## Materials and methods

### Samples

For proteomic research, a total of 12 primary ACC tumor tissues and their paired adjacent normal adrenocortical tissues were obtained from patients underwent resective operation at Shandong Tumor Hospital, China. For it is hard to obtain normal healthy adrenocortical tissues, we adopted normal adjacent adrenocortical tissues as a control of ACC in our proteomic studies. Fresh ACC tissue (3–5 mm in diameter) were obtained from the core part of cancer tissues without necrosis, and grossly normal adjacent tissues were taken from the resection margin of ACC tumors. Resected fresh tissues were first snap-frozen in liquid nitrogen, and stored at −80°C until use. For immunohistochemistry validation study, a total of 39 ACC and paired normal adrenocortical tissues, and 31 benign adrenocortical adenomas (ACA) were also obtained from Shandong Tumor Hospital. All the samples were histologically confirmed by two independent pathologists (D Mu and De Zhang). The study was started upon approved by the ethical committee of our institution, and samples were obtained with informed consent.

### Two dimensional electrophoresis (2-DE)

Frozen ACC and normal adjacent adrenocortical tissues were first homogenized using a sample grinding kit (GE Healthcare) with a lysis buffer (7 M urea, thiourea 2 M, 2% CHAPS, 18 mM DTT, 1 mM PMSF, 0.5% IPG buffer pH3~10NL), and then the extracts were centrifuged at 12,000 g, 4°C, for 1 hr. After the centrifugation, the supernatants were collected for 2-DE analysis. The protein concentration was determined using a 2D Quant kit (GE Healthcare).

We adopted a “sample pool” strategy in the comparative proteomic study as described previously [9]. Equal amount 500 μg of proteins extracted from ACC and normal adrenocortical tissues were pooled respectively, and diluted with rehydration buffer (8 M urea, 2% CHAPS, 0.5% IPG buffer, 40 mM DTT) for isoelectric focusing. After isoelectric focusing, the strips were first equilibrated with 130 mM DTT in equilibration buffer (6 M urea, 30% glycerol, 2% SDS, 50 mM Tris–HCl, pH 8.8), and then with 135 mM iodoacetamide in the same buffer. SDS polyacrylamide (SDS-Page) was performed with constant power (17 W/gel) at 20°C on an Ettan Dalt twelve system (GE healthcare). After the 2-DE, the gels were stained with Coomassie blue R350 and images were scanned for data analysis using Imagemaster 5.0 software package (GE healthcare).

### In-gel digestion and mass spectrometry identification

The gel pieces were first destained with 25 mM NH_4_CO_3_/50% ACN for 30 min, and dehydrated in 100% ACN for 10 min, and were then digested in 20 ng/μL sequencing grade-modified trypsin (Promega) overnight at 37°C. After extracted with 5% TFA/50% ACN, the peptides were resuspended in 3 μL of 0.1% TFA for mass spectrometry analysis. Protein identification was performed on 4700 Proteomic Analyzer MALDI-TOF-TOF mass spectrometer (Applied Biosystems) in a reflective mode. All mass spectrometry data were searched using the MASCOT search engine against a human subset of the Swiss-Prot database.

### Immunohistochemistry

Three proteins (calreticulin, prohibitin and HSP60) up-regulated in ACC identified in the proteomic study were selected for validation in an independent set of samples including 39 ACC, 31 ACA, and 39 normal adrenocortical tissues by immunohistochemistry (IHC). Briefly, after rehydration and deparaffinization, paraffin-embedded tissue slides were processed for antigen retrieval using heating in citrate buffer, and immunohistochemically stained with the rabbit polyclonal antibodies against human calreticulin (1:150 dilution; PTG-Lab) and prohibitin (1:150 dilution; PTG-Lab) and heat shock protein 60 (HSP60) (1:100 dilution; PTG-Lab). All these antibodies were widely used in IHC staining, and their specificity has been confirmed in many previous studies. Visualization was performed using a SP kit (Maixin-Bio, Fujian, China). For the negative controls, the primary antibody was replaced by rabbit IgG.

A semi-quantitative H score method was used to evaluate the results of IHC as described previously within minor modification [10]. Staining intensity was quantified using the image analysis program Leica Qwin V3, and were graded to four-scale (0–3), while the percentages of positive cells were scored into four-scale (0, no positive cells; 1, 1%–33% positive cells; 2, 34%–66%; and 3, 67%–100%). H score (ranging from 0 to 9) was calculated by multiplying staining intensity and the percentage of positive cells. The median H-score was used as a cut-off for classify low (< median H-score) and high (≥ median H-score) expression of each markers.

### Statistics

For proteomic study, spots with intensity changes greater than 2.0-fold were considered as differently expressed spots, and were excised from gels for mass spectrometry analysis. Gene Ontolgoy (GO) analysis was performed using MAS (Molecular Annotation System) 2.0 software (CapitalBio, China). For immunohistochemical results, the difference in H scores of ACC, ACA, and normal controls were compared with Mann–Whitney *t* test, and the correlation between biomarkers with the clinicopathological traits of ACC patients was evaluated with Chi-square or Student *t* test as appropriate. P value less than 0.05 was considered statistically significant.

## Results

### Comparative proteomic profiling between ACC and adjacent normal adrenocortical tissues

**Figure 1:**
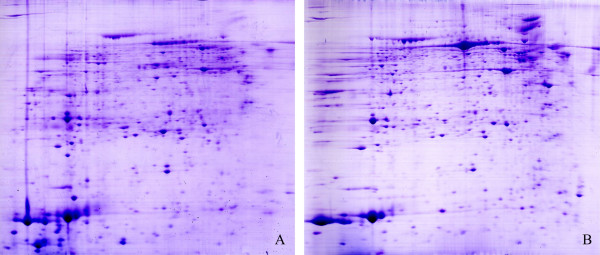
**Representative images of two-dimensional electrophoresis (2-DE).** 2-DE maps of adrenocortical carcinomas (**A**) and their paired adjacent normal adrenocortical tissues (**B**).

The 2-DE analyses were repeated in three replicas to guarantee the reproducibility of the results. As seen in Figure 1, representative gel images were selected for comparative proteomic analyses of ACC and their normal controls. According to the criteria established, spots with two fold variation between the two groups were defined as differentially expressed proteins. A total of 29 differentially expressed spots were successfully identified by mass spectrometry. Twenty proteins were identified as being up-regulated in ACC samples, compared with their corresponding proteins in normal adrenocortical tissues, while 9 proteins were identified to be down-regulated. The details of differently expressed proteins were summarized in Table 1.

**Table 1:** Differently expressed proteins between adrenocortical carcinomas and normal adrenocortical tissues in proteomic study

**Protein**	**Accession number**	**Molecular weight**	**pI**	**Number of matched peptides**	**Mascot score**	**Sequence coverage %**	**Fold**
Secretogranin-1	P05060	78343	5.02	24	245	39	2.3
Retinal dehydrogenase 1	P00352	55454	6.3	9	66	24	3.1
Prelamin-A/C	P02545	74379	6.57	26	234	37	2.2
Transitional endoplasmic reticulum ATPase	P55072	89949	5.14	31	383	45	4.1
Keratin, type I cytoskeletal 10	P13645	59020	5.13	11	84	20	2.8
Prohibitin	P35232	29843	5.57	10	148	51	3.8
Transgelin-2	P37802	22548	8.41	8	85	49	2.2
Selenium-binding protein 1	Q13228	52928	5.93	8	82	23	2.1
Actin, cytoplasmic 1	Q96HG5	42052	5.29	11	93	34	4.3
Glutathione S-transferase P	P09211	23569	5.43	12	139	60	2.7
Peroxiredoxin-2	P32119	22049	5.66	10	153	47	2.4
Rho GDP-dissociation inhibitor 1	P52565	23250	5.02	11	122	45	2.3
Elongation factor 1-beta	P24534	24919	4.5	10	113	46	3.3
Peroxiredoxin-1	Q06830	22324	8.27	11	165	62	3.2
14-3-3 protein epsilon	Q7M4R4	29326	4.63	11	111	41	5.1
60 kDa heat shock protein	P10809	61187	5.7	18	222	40	4.3
ATP synthase subunit d, mitochondrial	O75947	18537	5.21	9	119	59	3.8
Retinal dehydrogenase 1	P00352	55454	6.3	16	189	41	3.6
Calreticulin	P27797	48283	4.29	13	180	43	5.2
Aflatoxin B1 aldehyde reductase member 3	O95154	37582	6.67	15	148	48	2.6
T-complex protein 1 subunit beta	P78371	57794	6.01	15	140	43	−3.2
Elongation factor Tu, mitochondrial	P49411	49852	7.26	26	287	61	−4.1
Protein disulfide-isomerase	P07237	57479	4.76	17	171	37	−2.2
Vimentin	Q8N850	53676	5.06	12	85	33	−2.0
Serum albumin	Q9P157	71317	5.92	27	319	54	−3.7
Peroxiredoxin-2	P32119	22049	5.66	9	152	43	−2.8
Tropomyosin alpha-4 chain	Q9UCS3	28619	4.67	10	130	34	−2.5
Proteasome subunit beta type-2	P49721	22993	6.51	8	106	49	−3.3
Fibrinogen gamma chain	Q9UC63	52106	5.37	8	86	28	−3.3

### Gene Ontology (GO) analysis on the differently expressed proteins identified in proteomic study

As seen in Table 2, GO analysis on molecular function revealed that up-regulated proteins in ACC samples were enriched most in protein binding (Secretogranin-1, prelamin A/C, keratin 10, actin cytoplasmic 1, glutathione S-transferase P, elongation factor 1-beta, peroxiredoxin-1) and oxidoreductase activity (retinal dehydrogenase 1, peroxiredoxin-1, peroxiredoxin-2, aflatoxin B1 aldehyde reductase member 3).

**Table 2:** Gene Ontology (GO) analysis on up-regulated proteins in proteomics study

**GO term**	**Count**	**p-value**	**Gene symbol**
GO:0005515 protein binding	7	2.79E-08	CHGB;LMNA;KRT10;ACTB;GSTP1;EEF1B2;PRDX1
GO:0016491 oxidoreductase activity	4	2.79E-08	ALDH1A1;PRDX2;PRDX1;AKR7A3
GO:0051087 chaperone binding	2	4.17E-07	HSPD1;CALR
GO:0016887 ATPase activity	3	4.52E-07	VCP;HSPD1;ATP5H
GO:0051082 unfolded protein binding	2	1.33E-05	HSPD1;CALR
GO:0005524 ATP binding	3	3.47E-05	VCP;ACTB;HSPD1

### Expression of calreticulin, prohibitin and HSP60 in ACC, ACA and normal adrenocortical tissues by immunohistochemistry

**Figure 2:**
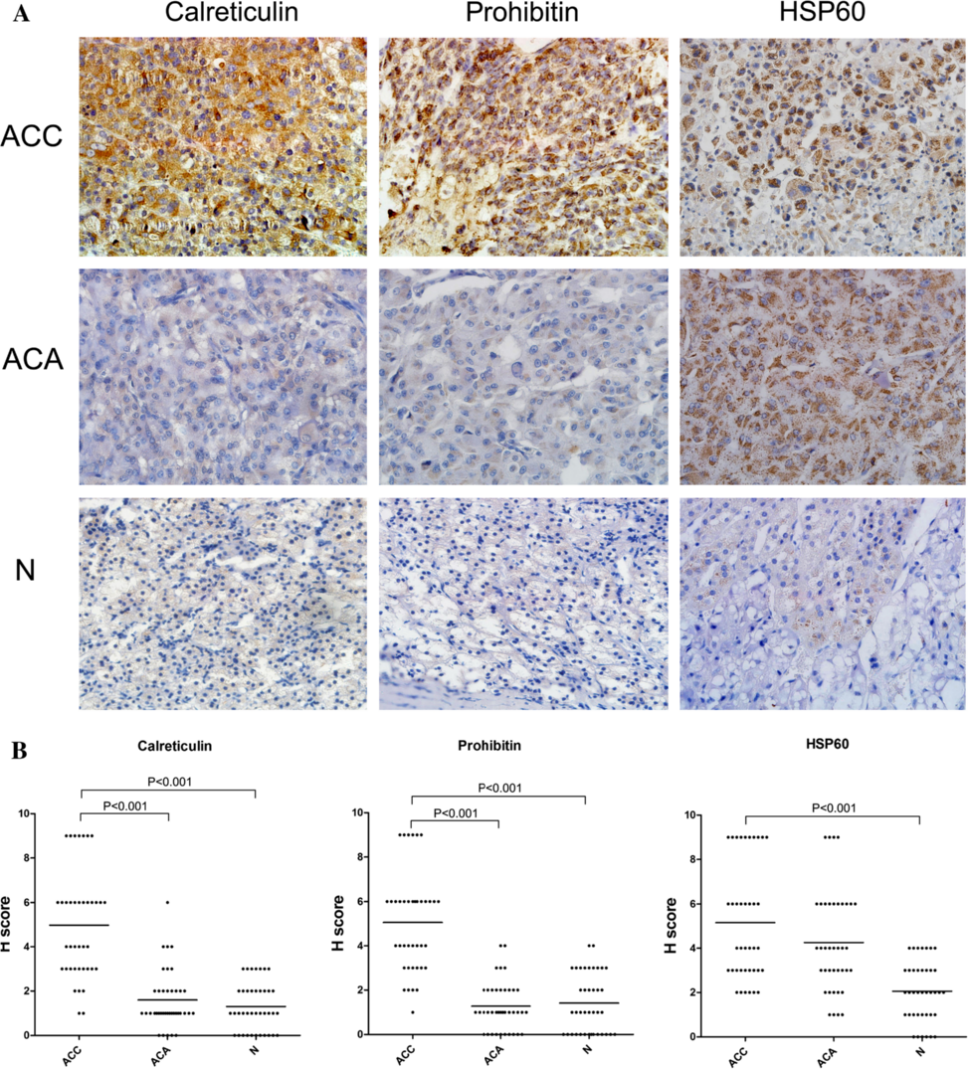
**Representative images of immunohistochemical results.** Expression of calreticulin, prohibitin and HSP60 in adrenocortical carcinomas (ACC), adrenocortical adenomas (ACA) and normal tissues (N) (**A**). Calreticulin and prohibitin but not HSP60 were overexpressed in ACC compared with adrenocortical adenomas (ACA) and normal tissues (**B**).

Three differentially expressed proteins, calreticulin, prohibitin and heat shock protein 60 (HSP60), which had not been reported in previous studies on ACC samples, were selected to be validated in a larger size of samples by immunishotchemistry. As seen in Figure 2, the expression levels (H score) of calreticulin, prohibitin and HSP60 were significantly higher in ACC samples than those in normal adrenocortical tissues, which are consistent with the findings in proteomic study. Furthermore, ACC tumors also demonstrated a higher expression level of calreticulin and prohibitin than ACA tumors, but the expression of HSP60 showed no significant difference between malignant and benign adrenocortical tumors. Negative staining with nonspecific rabbit IgG control was documented for each experiment (data not shown).

### Association of calreticulin and prohibitin expression with clinicopathological characteristics in ACC

According to the criteria for IHC evaluation, the median H-score of 6 was set as the cut-point to delineate low and high expression for calreticulin and prohibitin. The relationship between calreticulin and prohibitin expression with clinicopathological characteristics of ACC tumors was analyzed. As seen Table 3, no significant difference was observed between calreticulin and prohibitin expression with all the chinicopathological characteristics of ACC tumors, except that calreticulin overexpression was significantly associated with stages in ACC samples. High calreticulin expression was seen more frequently in advanced-stage ACC tumors than in early-stage cases (65.0% vs. 31.6%, P = 0.037).

**Table 3:** Association between calreticulin and prohibitin expression status with clinical charateristics in ACC

**Clinical characteristics**	**Total**	**Calreticulin expression**		**P**	**Prohibitin expression**		**P**
		**Low (n = 19)**	**High (n = 20)**		**Low (n = 18)**	**High (n = 21)**	
**Age (y, Mean ± SD)**		45 ± 17.4	37 ± 14.5	0.129	43 ± 18.6	39 ± 14.4	0.486
**Gender**							
Male	18	8	10	0.621	8	10	0.843
Female	21	11	10		10	11	
**Tumor location**							
Left	17	7	10	0.641	7	10	0.150
Right	19	10	9		8	11	
Bilateral	3	2	1		3	0	
**Primary tumor size**							
< 5.0 cm	5	2	3		1	4	
5.0 – 10.0 cm	28	16	12	0.181	14	14	0.454
> 10.0 cm	6	1	5		3	3	
**ENSAT stage**							
I- II	20	13	7	0.037	8	12	0.429
III- IV	19	6	13		10	9	
**Hormonal secretory status**							
Non-functioning tumor	21	12	9	0.256	11	10	0.399
Functioning tumor	18	7	11		7	11	

## Discussion

In this study, for the first time, we performed a 2-DE-based proteomic study to compare the protein profiling of ACC and normal adrenocortical tissues. A panel of protein markers were identified to be differently expressed. For only a few samples could be included in traditional proteomic studies, we adopted a “sample pool” strategy to increase the sample size. This strategy could also decrease the sample heterogeneity in some extent. To validate the results of proteomic analysis, we further validate three biomarkers calreticulin, prohibitin and HSP60 in a larger size of samples by immunohistochemestry. These proteins were selected for the following reasons: first, these biomarkers have a relatively high expression level in ACC, compared with normal adrenocortical tissues; secondly, previous studies have indicated that these genes are involved in the malignant progression of multiple cancers, but have not been evaluated in ACC; third, commercial antibodies for immunohistochemistry are available.

Consistent with our proteomic findings, we confirmed calreticulin, prohibitin and HSP60 overexpressed in ACC tumors than normal adrenocortical tissues. It has been suggested that the protein profiling of benign tumors partly resemble their malignant counterparts [11]. A candidate marker elevated in both ACC and ACA would lower their specificity in ACC diagnosis. Therefore, we further compared the expression of calreticulin, prohibitin and HSP60 in ACA and ACC. We found that HSP60 was overexpressed in both ACC and ACA, compared with their normal controls, which would lower its further utility as a candidate biomarker for ACC. Different from HSP60, ACC tissues had significantly higher expression levels of calreticulin and prohibitin than ACA, supporting their utility as specific biomarkers for ACC tumors.

Calreticulin was first identified as a Ca2+ binding protein, and has been implicated in many cellular functions and pathophysiological process such as cell adhesion, autoimmunity and heat shock [12,13]. Elevated expression of calreticulin has been reported in multiple cancers, and it is proposed that the upregulation of calreticulin seems to be induced by cellular stress from cancers [14]. Our results indicated that calreticulin correlated to tumor stage of ACC in clinical samples. However, the exact mechanisms for its increases in ACC are as yet undetermined.

Prohibitin has been shown to localize to mitochondria, and has been identified to be up-regulated in many cancers in previous studies [15]. However, experimental data about its role in tumorigenesis is conflicting. Several studies have suggested that prohibitin effects as a tumor suppressor [16], while other data indicated that prohibitin is required for the activation of several central signaling pathways related to carcinogenesis such as RAS-induced RAF-MEK-ERK activation [17]. Our findings supported that prohibitin upregualted in ACC tumors and its roles in ACC carcinogenesis deserves further investigation.

Except above three markers, we also identified other 17 up-regulated proteins in ACC, most of which (such as transitional endoplasmic reticulum ATPase and 14-3-3 protein epsilon) have been demonstrated to be involved in cancer carcinogenesis in other cancers in previous studies [18-20], but have not been reported to be associated with ACC. Therefore, these proteins might also be novel potential candidate markers for ACC, and deserves further investigation in the future.

## Conclusions

In this proteomic study, we identified and validated calreticulin and prohibitin overexpressed in ACC samples compared with their normal and benign counterparts, suggesting that these two markers are novel potential candidate biomarkers for ACCs. We proposed that the molecular mechanisms of calreticulin and prohibitin during ACC carcinogenesis also deserve further investigation in the future.

## Competing interests

The authors have declared no conflicts of interest.

## Authors’ contribution

YMS designed the study, performed the experiments, analyzed the data and drafted the manuscript; ZB, ZX, SXT, SXW, XS were responsible for the samples collection; WH and WB participated in proteomic studies; LW, PZ, GY participated in IHC; MD and ZD were responsible for the histological examination of specimens and IHC results evaluation and score. All the authors read and approved the final manuscript.
